# Effects of non-denatured whey protein supplementation combined with a Mediterranean-type diet on cognitive function and muscle health in sarcopenic older adults with cognitive impairment: a retrospective observational study

**DOI:** 10.3389/fnut.2026.1859189

**Published:** 2026-07-16

**Authors:** Lombardi Marta, Di Lauro Mariastella, Nasti Gilda, Caruso Marco, Petito Martina, Oliviero Nunzio, Di Martino Annabella, Piazza Roberto, Mangrella Mario, Guida Bruna, Chiurazzi Martina

**Affiliations:** 1 Department of Clinical Medicine and Surgery, Physiology Nutrition Unit, Federico II University of Naples, Naples, Italy; 2 Oncology Unit, Antonio Cardarelli Hospital, Naples, Italy; 3 Medical Affairs Department Italfarmaco SpA, Milan, Italy

**Keywords:** cognitive impairment, muscle strength, nutritional intervention, sarcopenia, whey protein supplementation

## Abstract

**Introduction:**

Population ageing has led to a rise in neurodegenerative disorders, driven by metabolic, inflammatory, and nutritional factors. The Mediterranean diet and adequate protein intake may help protect cognitive function and support muscle health. Sarcopenia is strongly associated with cognitive decline through the muscle–brain axis. This study evaluates the combined effects of a Mediterranean-type diet and whey proteins on cognition, body composition, and muscle strength in sarcopenic older adults.

**Methods:**

A total of sixty-eight older adults with sarcopenia and cognitive impairment were retrospectively enrolled and divided according to adherence to the nutritional intervention. Group A (*n* = 38) followed both a Mediterranean-type diet and whey protein supplementation, while Group B (*n* = 30) followed the diet alone and served as controls. Clinical, anthropometric, and body composition parameters were assessed at baseline, 3 and 6 months.

**Results:**

At 6 months, Group A was associated with significant improvements in body composition and functional outcomes compared with baseline. In particular, Body Cell Mass (BCM) increased, together with an improvement in right-hand grip strength, indicating enhanced muscle quality and strength. Muscle mass also increased, supporting better physical performance, as reflected by a reduction in perceived frailty (SARC-F score). Cognitive outcomes improved in both groups, with significant increases in MMSE scores, while executive function also improved in the intervention group, as shown by better TMT-A and TMT-B performance.

**Discussion:**

A Mediterranean-type diet combined with whey protein supplementation was associated with improvements in both muscle and cognitive function in sarcopenic older adults; however, due to the observational nature of the study, causal relationships cannot be established. These findings align with evidence on the benefits of protein intake for musculoskeletal health. Potential mechanisms include reduced inflammation and muscle–brain communication via myokines. The lack of resistance training may have limited long-term effects. Overall, results support combined nutritional strategies to improve ageing outcomes.

## 1 Introduction

Neurodegenerative disorders associated with population ageing represent a major public health challenge due to their impact on quality of life and the burden placed on healthcare systems (1). Among these conditions, Alzheimer’s disease, Parkinson’s disease and other forms of dementia are characterized by progressive cognitive decline, affecting memory, attention and executive functions, and ultimately leading to loss of functional independence (2). The increase in life expectancy observed in recent decades has been accompanied by a rise in the prevalence of these disorders, highlighting the need for effective preventive and therapeutic strategies (3). In addition to genetic factors, growing evidence suggests that metabolic, inflammatory and nutritional mechanisms contribute to both the onset and progression of neurodegenerative diseases (4). Ageing is associated with several biological changes that increase neuronal vulnerability, including hormonal alterations, impaired protein homeostasis, oxidative stress and a chronic low-grade inflammatory state known as inflammaging (5, 6). Although effective pharmacological strategies to prevent cognitive decline remain limited, nutrition has emerged as a key modifiable factor influencing disease risk and progression (7). Dietary patterns rich in antioxidant and anti-inflammatory compounds, such as omega-3 fatty acids, polyphenols and vitamins, may induce neuroprotective effects by modulating oxidative stress and inflammatory pathways (8). In this regard, adherence to the Mediterranean diet has been consistently associated with a lower risk of dementia and a slower rate of cognitive decline, likely due to its high content of bioactive compounds and overall nutritional quality (9, 10). Recent reviews further support the potential role of dietary interventions in promoting healthy cognitive ageing and reducing neurodegenerative risk (11, 12).

More recently, attention has shifted toward the role of skeletal muscle health in cognitive decline associated with ageing. Evidence suggests a close association between sarcopenia and cognitive impairment (13). Sarcopenia is an age-related condition characterized by progressive loss of muscle mass and strength, and is linked to increased disability and mortality (14). According to the European Working Group on Sarcopenia in Older People (EWGSOP2), reduced muscle strength is the primary indicator of the condition, while muscle mass and physical performance are used to confirm diagnosis and assess severity (15). Its prevalence increases with age, ranging from approximately 10 to 40%, and is higher in hospitalized and chronically ill populations (16). Individuals with sarcopenia show a higher risk of cognitive impairment, and more than half of patients with severe dementia are affected by this condition (17, 18). This association supports the concept of a “muscle–brain axis,” describing the bidirectional interaction between skeletal muscle and the central nervous system (19). Recent evidence suggests that this bidirectional interaction may also involve gut-derived signaling pathways, inflammatory mediators, and metabolic alterations associated with ageing, further suggesting a potential link between the muscle–gut axis and pathways implicated in cognitive decline and frailty (20).

Skeletal muscle acts as an endocrine organ, releasing myokines that influence metabolic and neurotrophic processes (21), while the central nervous system regulates muscle function through neuromuscular pathways. These interactions are sustained by shared pathophysiological mechanisms and may contribute to a cycle in which physical frailty and cognitive decline reinforce each other (22). Within this framework, nutrition plays a central role. Adequate protein intake is essential for maintaining muscle mass and counteracting anabolic resistance in older adults (23). Whey proteins are considered a high-quality source due to their high biological value and rapid absorption. They are rich in essential amino acids, particularly leucine, which stimulates muscle protein synthesis through activation of the mTOR pathway (24, 25). In addition, native whey proteins contain cysteine, a precursor of glutathione, one of the main intracellular antioxidants, whose levels decline with ageing and in neurodegenerative conditions (26). For this reason, whey protein supplementation may support both muscle maintenance and brain health (27). Based on these considerations, the present study aims to investigate the associations between a Mediterranean-type dietary pattern combined with non-denatured whey protein supplementation and cognitive performance, assessed using the Trail Making Test (TMT) parts A and B and the Mini-Mental State Examination (MMSE), as well as body composition and muscle strength in sarcopenic older adults with cognitive impairment.

## 2 Methods

### 2.1 Study design

The study protocol was approved by the Ethics Committee of the Federico II University Medical School of Naples (EC approval code: [29/2025]. Due to the retrospective nature of the study, data were collected from medical records and analysed anonymously. A total of 68 older adults of both sexes (32 men and 36 women; 70.4 ± 10.8 years) with a diagnosis of sarcopenia according to the European Working Group on Sarcopenia in Older People 2 (EWGSOP2) criteria and mild-to-moderate cognitive impairment, identified by the Mini-Mental State Examination (MMSE score ≤ 24), were retrospectively identified among patients attending the outpatient clinic of the Departmental Program “Diet Therapy in Transplantation and Chronic Renal Failure” at the School of Medicine, University of Naples “Federico II” and enrolled from September 2024 to November 2025. Demographic and clinical characteristics, biochemical parameters, pharmacological treatments, anthropometric measurements, body composition, and physical activity levels were retrieved from clinical records at baseline and at approximately 3 and 6 months of follow-up. As part of routine clinical practice, all patients with sarcopenia were prescribed an individualized Mediterranean-type dietary plan tailored to estimated energy and protein requirements, together with oral supplementation with non-denatured whey proteins with high biological value (Prother®, 10 g/day). Adherence to protein supplementation was assessed during follow-up visits. Patients were initially prescribed the same dietary intervention, including a Mediterranean-type diet combined with oral nutritional supplementation; however, adherence to protein supplementation varied during follow-up. For the purpose of the present analysis, participants were therefore classified according to observed adherence as documented in clinical records. The first group (Group A, *n* = 38) included patients who adhered to both the dietary prescription and protein supplementation over the 3 and 6-month follow-up. The second group (Group B, *n* = 30) comprised patients who followed the prescribed dietary regimen but did not take the protein supplement. Therefore, group B should be interpreted as a non-adherent group with respect to protein supplementation. Exclusion criteria included the presence of chronic kidney disease, active malignancies, severe osteoporosis, advanced liver failure, and gastrointestinal disorders that could significantly impair digestive or nutrient absorption processes.

### 2.2 Study protocol

Data were obtained from routine clinical assessments performed at baseline (T0) and at follow-up visits at approximately 3 (T1) and 6 months (T2). To evaluate their nutritional status, several anthropometric measurements were collected, including weight (measured using a Seca GmbH & Co KG scale, Hamburg, Germany), height (using a wall-mounted stadiometer with precision to the nearest 0.1 cm) and body mass index (BMI) (28). Additional body circumferences (waist, hip, arm, and calf) were also recorded using a flexible anthropometric tape (29). Body composition had been assessed by bioelectrical impedance analysis (BIA) using a tetrapolar device (RJL 101; Akern SRL, Florence, Italy) with a single-frequency measurement (50 kHz). BIA with a single frequency provides the best information at a body level, because it minimizes frequency-dependent errors and variability of electric flow paths. Measurements were performed in the supine position after a short period of rest, according to standard clinical practice in our outpatient setting. Patients were assessed in a fasting state and in the absence of clinical signs of over hydration or dehydration, under routine and comparable real-life conditions across participants. Body Cell Mass (BCM) was considered a clinically relevant body composition parameter because it reflects the metabolically active component of fat-free mass, including the intracellular compartment involved in protein metabolism and energy production. In older adults, BCM is regarded as a sensitive marker of nutritional and functional status, capable of capturing early changes in cellular and muscle quality that may not be evident through conventional anthropometric measures. For this reason, BCM is commonly used in clinical nutrition and sarcopenia research as an indicator of functional tissue mass (30).

Muscle strength was evaluated using a handgrip dynamometer (78,010; Lafayette Instrument Company, Lafayette, IN, United States). Handgrip strength was measured in the standing position, with the arm along the body and the elbow fully extended, using the dominant hand. Three attempts were performed, and the mean value was recorded for the analysis. Reduced muscle strength was defined according to commonly adopted cut-offs (<27 kg in men and <16 kg in women) (31). Dietary intake was assessed using weekly Food Frequency Questionnaires (FFQs), which recorded both the frequency and quantity of food and beverage consumption (32). Screening for sarcopenia was performed using the SARC-F questionnaire, consisting of five items scored from 0 to 2 each; a total score ≥4 was considered predictive of sarcopenia (33). Cognitive function had been evaluated using the Mini-Mental State Examination (MMSE) and the Trail Making Test (TMT) parts A and B during routine clinical practice. A clinically meaningful change for the MMSE was defined as a difference of at least 2 points, which has been reported in the literature as the minimum threshold for detecting a significant change in cognitive-status (34). For the TMT-A and TMT-B, a clinically relevant improvement was defined as a reduction of at least 30 s in completion time compared with baseline, in line with findings reported in studies involving populations with mild cognitive impairment (35). Additionally, blood parameters, including blood glucose (Gly), insulin, total cholesterol (COL-tot), HDL cholesterol (Col-HDL), LDL cholesterol (Col-LDL), triglycerides (Try), and transaminases (GOT and GPT), were measured as part of routine clinical monitoring.

### 2.3 Dietary treatment and compliance

A personalized dietary plan had been prescribed for each patient in accordance with the Italian Dietary Reference intakes for nutrients and energy based on the recommended values for adults aged 65–74 and ≥75 years, as appropriate (LARN, V Revision, 2024). All participants were advised to follow a Mediterranean dietary pattern adapted to their individual energy requirements. Adherence to the Mediterranean dietary pattern was monitored during follow-up visits through structured dietary anamnesis, focusing on habitual intake of key food groups such as fruit, vegetables, legumes, whole grains, olive oil, and fish. Daily energy needs were estimated starting from basal metabolic rate calculated using the revised Harris–Benedict equation and then adjusted according to the subject’s level of physical activity. In underweight individuals, energy intake was set at approximately 35 kcal/kg/day. Protein intake was set at 1.5 g per kilogram of ideal body weight per day and distributed across the main daily meals (breakfast, snacks, lunch, and dinner) in order to support muscle mass maintenance and increase (36). In addition to the dietary intervention prescribed to all patients, oral nutritional supplementation with Prother®, a food for special medical purposes based on undenatured whey proteins with high biological value (DIF16WPI), was also recommended at a dose of 10 g per day. Patients in Group A were those who adhered to the prescribed protein supplementation during follow-up. Adherence to protein supplementation was assessed separately based on patient self-report and clinical documentation during follow-up visits. Prother®, classified as a Food for Special Medical Purposes, is commonly used in clinical practice for the nutritional management of sarcopenia due to its high protein concentration (92.5%) and its production through low-temperature microfiltration, which helps preserve the native structure and biological activity of whey proteins. Each 10 g dose of Prother® provides 9.25 g of protein and is characterized by a cysteine-rich profile (2.7%). The average nutritional composition per 10 g is as follows: energy 36.4 kcal, fats ≤0.1 g, carbohydrates < 0.12 g, sugars < 0.12 g, salt 0.0375 g, calcium 50 mg, potassium 50 mg, sodium 15 mg, and phosphorus 25 mg. No common adverse effects have been reported for this product; however, possible gastrointestinal symptoms or headache were recorded in the clinical documentation during follow-up visits.

### 2.4 Statistical analysis

**Figure 1:**
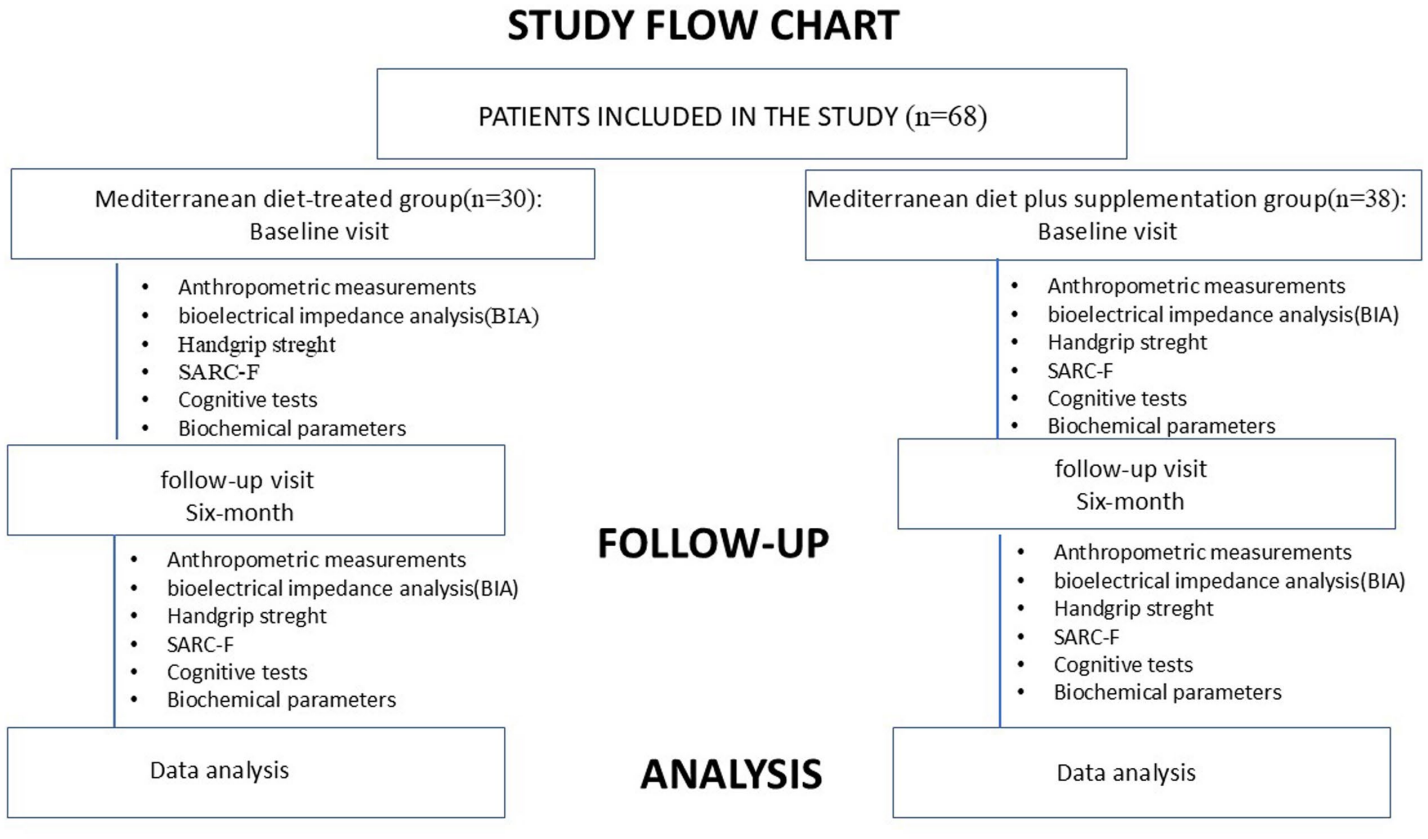
Study flow chart showing patient allocation, baseline and six-month follow-up assessments, and data analysis.

For categorical variables, absolute numbers and frequencies (%) are shown. The Kolmogorov–Smirnov test was used to check normality. Normally distributed variables are presented as the means ± standard deviations (SDs). Non normally distributed variables are expressed as the median and interquartile range. For comparisons between baseline data and follow-up data, normally distributed data were analysed via paired t tests. For comparisons between independent groups, Student’s t test and chi-square tests were used. The sample size was calculated using G*Power software (paired *t*-test, two-tailed, *α* = 0.05, power = 0.95). Based on an expected effect size of dz. = 0.41, the required total sample size was estimated at 78 participants. A univariate General Linear Model (GLM) was performed to evaluate the effect of treatment on changes in the studied parameters. Age, sex, antihypertensive therapy, lipid-lowering therapy, and pantoprazole use were included as covariates. Homogeneity of variances was assessed using Levene’s test. All the statistical analyses were performed via SPSS 29.0 (SPSS Inc., Chicago, IL, United States). The statistical significance was set at *p* < 0.05, Figure 1.

## 3 Results

**Figure 2:**
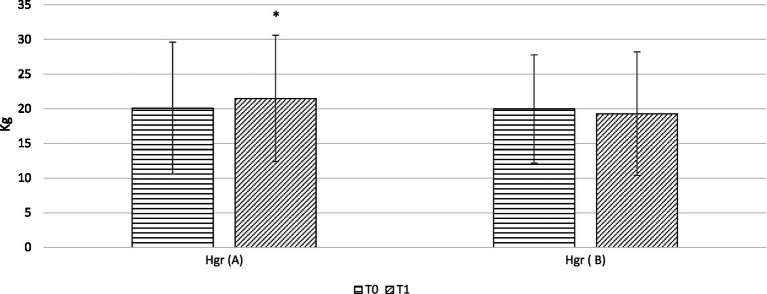
Hgr (Kg) of group **(A)** and **(B)** after 6 months of treatment. Data are reported as mean ± SD. *p < 0.05 T2 vs. T0.

**Figure 3:**
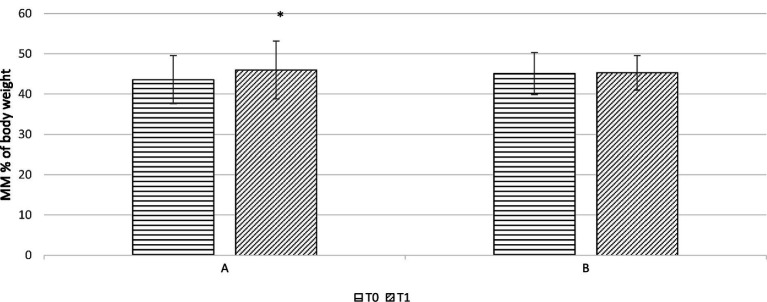
MM (% of body weight) of group **(A)** and **(B)** after 6 months of treatment. Data are reported as mean ± SD. *p < 0.05 T2 vs. T0.

**Figure 4:**
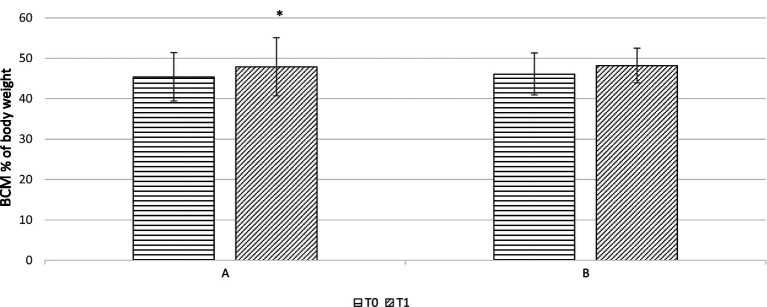
BCM (% of body weight) of group **(A)** and **(B)** after 6 months of treatment. Data are reported as mean ± SD. *p < 0.05 T2 vs. T0.

**Figure 5:**
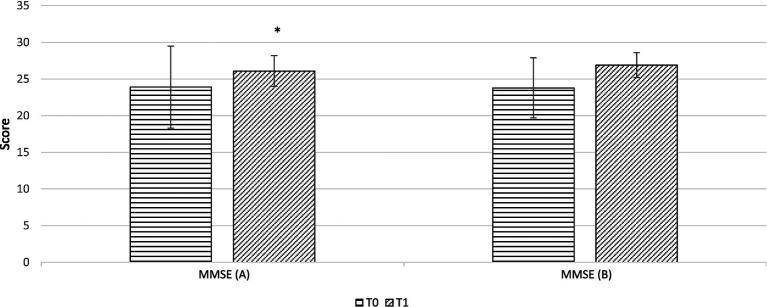
MMSE Test of group **(A)** and **(B)** after 6 months of treatment. Data are reported as mean ± SD. *p < 0.05 T2 vs. T0.

**Figure 6:**
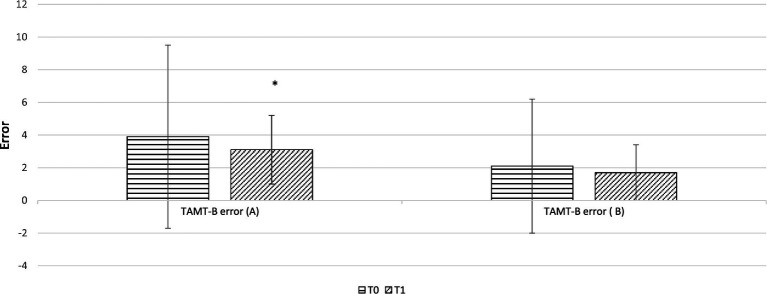
TAMT-B error Test of group **(A)** and **(B)** after 6 months of treatment. Data are reported as mean ± SD. *p < 0.05 T2 vs. T0.

**Figure 7:**
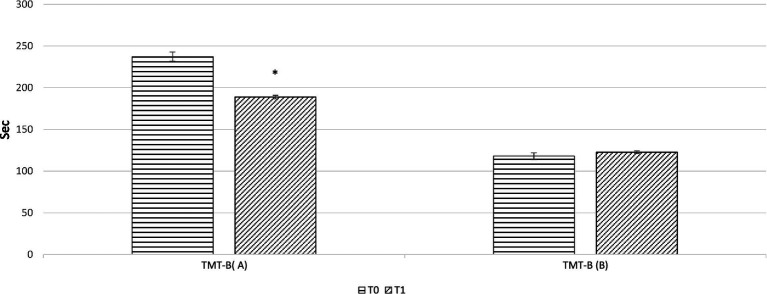
TMT-B Test of group **(A)** and **(B)** after 6 months of treatment. Data are reported as mean ± SD. *p < 0.05 T2 vs. T0.

The baseline demographic characteristics, anthropometric measurements, body composition, and metabolic parameters of the study population (*n* = 68; 47.1% males; mean age 70.4 ± 10.8 years; mean BMI 21.6 ± 3.2 kg/m^2^) are reported in Table 1. No significant differences in baseline demographic characteristics, anthropometric measurements, body composition, metabolic parameters, or major comorbidities were observed between the two groups. Table 2 reports the 6-month results compared with baseline for both groups. Significant improvements in anthropometric parameters and body composition were observed only in Group A, which had been prescribed a Mediterranean diet combined with oral supplementation of high-biological-value non-denatured whey protein (Prother®, 10 g/day). Specifically, Group A showed increases in body weight (55.1 ± 9.1 vs. 54.4 ± 9.5 kg, T2 vs. T0, *p* < 0.03), BMI (21.7 ± 3.1 vs. 21.4 ± 3.3 kg/m^2^, T2 vs. T0, *p* < 0.03), and right-hand grip strength (21.5 ± 7.8 vs. 20.1 ± 8.7 kg, T2 vs. T0, *p* < 0.03) (Figure 2). Group A also showed improvements in extracellular water (ECW) percentage (51.2 ± 4.8% vs. 53.7 ± 4.3%, T2 vs. T0, *p* < 0.02), and in muscle mass both in kilograms (25.6 ± 7.0 vs. 23.8 ± 5.7 kg, T2 vs. T0, *p* < 0.005) (Figure 3) and as a percentage (46.0 ± 7.2% vs. 43.6 ± 6.0%, T2 vs. T0, *p* < 0.02). Changes in left-hand grip strength were not significant. Waist circumference also increased significantly (84.1 ± 9.1 vs. 81.4 ± 11.0 cm, T2 vs. T0, *p* < 0.01) only in Group A, whereas hip, arm, and calf circumferences did not change notably in both groups. Body composition analysis revealed that Group A showed an increase in Body Cell Mass (BCM) percentage (47.9 ± 5.2 vs. 45.4 ± 4.8%, T2 vs. T0, *p* < 0.0001), indicating improved cellular and metabolically active mass (Figure 4). No significant changes were observed in total body water (TBW) or fat mass, although Group A showed a small increase in fat mass in kilograms (14.5 ± 5.5 vs. 13.0 ± 5.5 kg, T2 vs. T0, *p* < 0.05). In Group A, Muscle Mass Index (IMM) and Muscle Mass (MM, % and kg) also increased significantly (8.1 ± 2.3 vs. 7.4 ± 1.0 kg/m^**2**^ (IMM), 25.6 ± 7 vs. 23.8 ± 5.7 (MM Kg), 46 ± 7,2 vs. 43,6 ± 6,0 (MM %), T2 vs. T0, *p* < 0.05). Regarding functional and cognitive outcomes, Group A showed improvements in SARC-F score (1.2 ± 1.3 vs. 1.7 ± 1.8, T2 vs. T0, *p* < 0.04), indicating reduced perceived frailty. MMSE scores increased significantly in both groups (Group A: 23.9 ± 5.9 vs. 26.1 ± 2.1; Group B: 23.8 ± 4.1 vs. 26.9 ± 1.7; T2 vs. T0, *p* < 0.05) (Figure 5). The TMT-A test showed a significant reduction in errors in Group A (0.4 ± 3.3 vs. 0.74 ± 1.1, T2 vs. T0, *p* < 0.05), with no significant change in the time required to complete the test (seconds). In contrast, the TMT-B test showed significant improvements in both completion time (188.8 ± 123.1 vs. 237.2 ± 156.3 s, T2 vs. T0, *p* < 0.05) and errors (3.1 ± 3.4 vs. 3.9 ± 3.5, T2 vs. T0, *p* < 0.04) (Figures 6, 7), observed only in Group A. No additional improvements were observed at 12 months compared with 6 months (data not shown). Furthermore, Blood parameters, including blood glucose (Gly), insulin, total cholesterol (COL-tot), HDL cholesterol (Col-HDL), LDL cholesterol (Col-LDL), triglycerides (Try), and transaminases (GOT and GPT), were monitored throughout the entire duration of the study and remained stable over time (data not shown). In the end, for Hgr and MMSE test, a significant effect of the treatment group emerged, [*F*(1,45)=5.6, *p* = 0.024, η^2^p = 0.109, Hgr; *F*(1,45)=6.3, *p* = 0,016, η^2^p = 0.128, MMSE] with a greater improvement observed in the supplement-treated group compared with the diet-only group. Additionally, both sex (*p* = 0.004) and age (*p* = 0.008) were significantly associated with changes in Hgr.

**Table 1:** Demographic, anthropometric, and nutritional characteristics of the 68 patients in the study.

Parameters	Values
Sex (*n*, %)	M 32 (47.1%)
Age, years	70.4 ± 10.8
BMI, kg/m^2^	21.6 ± 3.2
Weight, Kg	74.6 ± 11.7
WC, cm	81.4 ± 10.3
IMM, Kg	7.4 ± 0.9
BCM, % of body weight	45.6 ± 4.3
TBW, % of body weight	60.5 ± 6.6
ECW, % of total body water	53.4 ± 3.9
FM, % of body weight	24.7 ± 8.3
FFM, % of body weight	75.3 ± 8.3
MM, % of body weight	44.0 ± 5.8
Lipid-lowering agents (*n*, %)	21 (30%)
Antihypertensive therapy (*n*, %)	48 (70%)
Proton pump inhibitors (*n*, %)	4 (5%)

Continuous variables are expressed as the mean ± SD. *p < 0.05. Categorical variables are expressed as numbers and percentages. BMI, body mass index; WC, waist circumference; FM, fat mass; FFM, fat-free mass; ECW, Extracellular water; TBW, Total body water; BCM, Body cell mass; MM, Muscle mass, IMM, Muscle mass index.

**Table 2:** Demographic, anthropometric, and nutritional characteristics of group A and B after 6 months of treatment.

Parameters	GROUP A (*n* = 38)		GROUP B (*n* = 30)	
	T0	T2	T0	T2
Male, *n*° (%)	20 (52.6)	20 (52.6)	12 (40.0)	12 (40.0)
Lipid-lowering agents (*n*, %)	11 (28.9%)	11 (28.9%)	10(33.3%)	10 (33.3%)
Antihypertensive therapy (*n*, %)	28 (73.7%)	28 (73.7%)	20 (66.7%)	20 (66.7%)
Proton pump inhibitors (*n*, %)	2(5.3%)	2(5.3%)	2 (6.7%)	2 (6.7%)
Age, years	71.0 ± 7.4	71.0 ± 7.4	65.6 ± 15.4	65.6 ± 15.4
Weight, kg	54.4 ± 9.5	55.1 ± 9.1*	55.6 ± 7.8	55.3 ± 8.9
BMI, kg/m^2^	21.4 ± 3.3	21.7 ± 3.1*	21.2 ± 3.3	21.4 ± 3.5
Hgr, Kg	20.1 ± 8.7	21.5 ± 7.8*	20.0 ± 5.9	19.3 ± 4.7
Hgl, Kg	18.3 ± 8.1	18.8 ± 6.84	17.7 ± 5.2	17.8 ± 4.7
WC, cm	81.4 ± 11.0	84.1 ± 9.2*	82.7 ± 9.8	85.9 ± 11.3
HC, cm	91.5 ± 7.1	92.1 ± 6.6	92.2 ± 6.6	92.7 ± 6.9
ACr, cm	24.9 ± 3.2	25.0 ± 2.3	24.5 ± 2.5	24.7 ± 3.4
ACl, cm	25.7 ± 2.9	25.7 ± 2.3	25.5 ± 2.5	25.2 ± 23.7
Calf, cm	32.8 ± 2.7	32.8 ± 2.5	33.2 ± 2.9	34.0 ± 2.1
IMM, kg/m^2^	7.4 ± 1.0	8.1 ± 2.3*	7.5 ± 0.7	7.4 ± 0.7
BCM, % of body weight	45.4 ± 4.8	47.9 ± 5.2*	46.1 ± 2.9	48.2 ± 3.4
TBW, % of body weight	60.3 ± 6.7	51.2 ± 4.8	60.7 ± 6.4	59.1 ± 5.9
ECW, % of total body water	53.7 ± 4.3	51.2 ± 4.8*	52.8 ± 2.8	50.8 ± 3.3
FM, Kg	13.3 ± 6.1	13.3 ± 6.4	13.0 ± 5.5	14.5 ± 5.5
FM, % of body weight	25.3 ± 8.6	24.0 ± 9.3	23.3 ± 8.0	25.3 ± 6.0
FFM, kg	40.3 ± 7.6	41.8 ± 8.5	41.6 ± 5.3	41.0 ± 4.9
FFM, % of body weight	74.7 ± 8.6	75.9 ± 9.3	76.9 ± 7.7	74.7 ± 6.9
MM, kg	23,8 ± 5,7	25,6 ± 7*	24.4 ± 3.5	25.0 ± 3.5
MM, % of body weight	43,6 ± 6,0	46 ± 7,2*	45.1 ± 5.2	45.3 ± 4.3

Parameters	COGNITIVE TESTS			
	GROUP A (*n* = 38)		GROUP B (*n* = 30)	
	T0	T2	T0	T2
MMSE, score	23.9 ± 5,6	26.1 ± 2.1*	23.8 ± 4.1	26.9 ± 1.7*
TMT—A, sec	72.7 ± 67.8	62.7 ± 34.8	57.9 ± 25.3	53.2 ± 20.8
TAMT—A, error	0.74 ± 1.1	0.4 ± 3.3*	1.0 ± 2.3	0.4 ± 0.5
TMT—B, sec	237.2 ± 156.3	188.8 ± 123.1*	117.9 ± 47.9	122.6 ± 72.0
TAMT—B, error	3.9 ± 3.5	3.1 ± 3.3*	2.1 ± 2.4	1.7 ± 1.0
SARC—F, score	1.7 ± 1.8	1.2 ± 1.3*	0.7 ± 0.7	0.7 ± 0.8

Continuous variables are expressed as the mean ± SD. Categorical variables are expressed as numbers and percentages. *p < 0.05. BMI, body mass index; WC, waist circumference; HC, hip circumference; ACr, Arm circumference right; ACl, Arm Circumference left; FM, fat mass; FFM, fat-free mass; ECW, Extracellular water; TBW, Total body water; BCM, Body Cell Mass; MM, Muscle Mass; IMM, Muscle mass index; MMSE, Mini-mental state examination; TMT—A (sec), Trail making test—Part A (seconds); TAMT—A (errori), Errors in Trail Making Test—Part A; TMT—B (sec), Trail Making Test—Part B (seconds); TAMT—B (errori), Errors in trail making test—Part B; SARC-F, Strength, Assistance in walking, Rise from a chair, Climb stairs, Falls.

## 4 Discussion

The present study evaluated whether a Mediterranean-type dietary approach combined with non-denatured whey protein supplementation (Prother®, 10 g/day) could improve muscle mass, physical performance, and cognitive function in sarcopenic older adults. Participants in the intervention group showed significant longitudinal changes compared with baseline, suggesting a possible association between this combined nutritional strategy and both musculoskeletal and cognitive domains of ageing. These findings are consistent with previous evidence indicating that protein supplementation can improve muscle-related outcomes in older adults with sarcopenia. In line with this, BCM, a marker of metabolically active cell mass, also increased, supporting the observed improvements in nutritional and functional status. Randomized controlled trials and meta-analyses have reported that whey protein supplementation is associated with increases in muscle mass index, lean mass, and physical performance measures such as gait speed and handgrip strength, particularly when combined with resistance training (37, 38). However, the effects are not uniform across all functional outcomes, and the greatest benefits are generally observed when nutritional interventions are integrated with structured exercise programs (39, 40). This is consistent with recent interventional studies showing that combined exercise and protein-based strategies may exert synergistic effects on muscle function and physical performance in older adults with sarcopenia (39, 41). Although no resistance training was included in our routine clinical management, the improvements observed in muscle mass and strength may partly explain the cognitive benefits detected. Interestingly, waist circumference also increased in the intervention group. Although this finding may appear counterintuitive, it occurred alongside improvements in BCM, muscle mass, and handgrip strength. In this context, the increase in waist circumference may reflect overall improvements in nutritional status and body composition rather than a selective increase in abdominal fat. Skeletal muscle is increasingly recognized as an endocrine organ capable of releasing myokines that may influence neuroinflammation, synaptic plasticity, and brain metabolism (42, 43). Likewise, adequate protein intake has been proposed to be involved in biological pathways such as oxidative stress and chronic low-grade inflammation, which have been associated with neurodegenerative processes (41). Emerging evidence suggests possible links between muscle-derived signaling pathways, inflammatory status, and neurodegeneration. However, these mechanisms were not assessed in the present study and should therefore be interpreted with caution. However, MMSE scores improved in both groups, suggesting that factors beyond protein supplementation, including adherence to the Mediterranean-type dietary pattern, may have contributed to the observed cognitive changes (29). Our results are also in line with observational and longitudinal studies suggesting that higher protein intake is associated with a slower rate of cognitive decline in older adults (44). Nevertheless, direct evidence linking whey protein supplementation specifically to cognitive outcomes remains limited, and the present findings should therefore be interpreted with caution (45, 46). An important aspect of this study is that adherence to both the Mediterranean diet and protein supplementation was evaluated based on routinely collected clinical data, reflecting real-world nutritional management in older adults with sarcopenia. Interestingly, no additional improvements were observed at 12 months compared with 6 months, suggesting that the initial benefits may plateau over time in the absence of additional stimuli such as resistance training. This finding supports the idea that multimodal approaches combining nutrition and exercise are likely necessary to maximize long-term benefits. Several implications for future research emerge from these results. Larger studies are needed to confirm these findings and assess their generalizability in more heterogeneous populations. The inclusion of structured resistance training protocols would help clarify potential synergistic effects between dietary and exercise interventions. In addition, the use of more comprehensive neuropsychological assessments, along with functional outcomes such as activities of daily living, would allow a better evaluation of the clinical relevance of cognitive changes. Finally, mechanistic studies investigating inflammatory markers, neurotrophic factors, and muscle-derived signaling molecules could further elucidate the pathways underlying the muscle–brain interaction. The present findings should be interpreted in light of several limitations, including the retrospective observational design, the relatively small sample size, the absence of randomization, and potential residual confounding related to adherence behavior. In addition, repeated administration of MMSE and TMT may have introduced practice effects, which could have contributed to part of the observed cognitive improvement. Finally, multiple comparisons were performed across several anthropometric, cognitive, and body composition outcomes, which may increase the risk of type I error inflation. Larger prospective randomized studies are warranted to confirm these preliminary findings. In conclusion, this study adds to the growing evidence that targeted nutritional strategies combining whey protein supplementation with a Mediterranean dietary pattern may improve both musculoskeletal and cognitive health in sarcopenic older adults. These findings support the inclusion of tailored nutritional interventions as part of multimodal strategies aimed at preserving physical function and cognitive performance during ageing.

## Data Availability

The original contributions presented in the study are included in the article/supplementary material, further inquiries can be directed to the corresponding author.
